# Locally Embedding Autoencoders: A Semi-Supervised Manifold Learning Approach of Document Representation

**DOI:** 10.1371/journal.pone.0146672

**Published:** 2016-01-19

**Authors:** Chao Wei, Senlin Luo, Xincheng Ma, Hao Ren, Ji Zhang, Limin Pan

**Affiliations:** Beijing Institute of Technology, Beijing, 10081, China; Jiangnan University, CHINA

## Abstract

Topic models and neural networks can discover meaningful low-dimensional latent representations of text corpora; as such, they have become a key technology of document representation. However, such models presume all documents are non-discriminatory, resulting in latent representation dependent upon all other documents and an inability to provide discriminative document representation. To address this problem, we propose a semi-supervised manifold-inspired autoencoder to extract meaningful latent representations of documents, taking the local perspective that the latent representation of nearby documents should be correlative. We first determine the discriminative neighbors set with Euclidean distance in observation spaces. Then, the autoencoder is trained by joint minimization of the Bernoulli cross-entropy error between input and output and the sum of the square error between neighbors of input and output. The results of two widely used corpora show that our method yields at least a 15% improvement in document clustering and a nearly 7% improvement in classification tasks compared to comparative methods. The evidence demonstrates that our method can readily capture more discriminative latent representation of new documents. Moreover, some meaningful combinations of words can be efficiently discovered by activating features that promote the comprehensibility of latent representation.

## Introduction

The performance of document analysis and processing systems based on machine learning methods, such as classification[1][2], clustering[3][4], content analysis[5], textual similarity[6], and statistical machine translation (SMT)[7], is heavily dependent on the level of document representation (DR), as different representations may capture and disentangle different degrees of explanatory ingredients hidden in the documents[8]. From the view of bag of words model, a document is typically represented via a point or vector in space whose dimensions (features) represent certain aspects of the document, such as observed variable (i.e., word or phrase). The vector space model (VSM) presents document vectors with different term-weighting approaches to observed words, such as *tf-idf*. However, such a representation ignores the semantic relations between words; due to the phenomena of polysemy and synonymy, observed words are highly correlated.

Some attempts to extract meaningful latent representations in text corpora have been proposed to overcome the limitation of the VSM. Latent semantic indexing (LSI)[9] decomposes the original vector space and project documents onto a subspace that captures the semantic relations between words. Several unsupervised topic models that have shown superior performance over LSI, including probabilistic latent semantic analysis (PLSA)[10] and latent Dirichlet allocation (LDA)[11]. These models conceptualize each document as a list of mixing proportions of latent topics, thus interpreting each topic as a distribution of vocabulary[12]. Such models can reveal latent topic representations by implicitly preserving the statistical relation of word co-occurrence[13]. Some supervised topic models, such as supervised latent Dirichlet allocation (SLDA)[14] and MedLDA[15], use side information (i.e., category labels) to improve the predictive power of latent document representations. Neural networks can also capture meaningful latent document representations (i.e., distributed representations) with deep learning techniques, including autoencoders[16], restricted Boltzmann machines (RBMs)[17], neural topic models (NTMs)[18] and document neural autoregressive distribution estimators (DocNADEs)[19]. These methods use the word count vector as input and synthesize the input through different hidden layers of various deep neural networks. Similar to topic models, such hidden layers can provide low-dimensional document representations. In essence, topic models and neural networks are embedded with latent factors or topics, preserving the salient statistical structure of intra-documents[19]. Although they represent an improvement for DR, such methods take a global perspective on document space as Euclidean, assuming that all documents are non-discriminatory and indicating that the latent representation is dependent on all other documents. Thus, they cannot provide more discriminative representation. However, recent studies[20][21][22] have shown that natural observations, such as documents and images, concentrate in the vicinity of a smooth lower-dimensional manifold, unable to fill up the Euclidean space. Consequently, better representation of the latent document semantics depends on modeling the local document relationship within a neighborhood.

Several topic models that consider the geometrical structure of documents were proposed, such as Laplacian probabilistic latent semantic indexing (LapPLSI)[23], locally consistent topic modeling (LTM)[24], and the discriminative topic model (DTM)[25]. Such models provide topic distributions that concentrate around the document manifold and are more effective than PLSA and LDA in text clustering and classification. However, they rely on the explicit construction of neighborhood graphs and fail to provide a definite mapping function between latent representations and manifold. Regarding neural networks, two autoencoder variants–the denoising autoencoder (DAE)[26] and the contractive autoencoder (CAE)[27]–demonstrate a promising ability to learn robust latent representations, which could induce the “intrinsic data structure”. However, these methods consider self-reconstruction without considering valuable class label information. Thus, learned representations may not be sufficiently reasonable in terms of similarity measurements because the representations of inter-class neighbors may congregate in the latent space[28].

Our main contribution is that taking a localized approach, we propose a semi-supervised manifold-inspired method known as the *locally embedding autoencoder* (LEAE). Given an input vector regarding the bag of words representation of each document, LEAE extracts meaningful low-dimensional latent representation via a regularized autoencoder, assuming that the latent representation of each document is strongly associated with its neighbors. Specifically, based on locally Euclidean hypothesis, we first select the neighbors belonging to the same categories according to the Euclidean distance in observations space. Then, through the encode-decode process of autoencoder, we synthesize a same size vector with input vector as output to approximate the bag of words representation of input and neighbors. Finally, we extract hidden layer value of autoencoder as low-dimensional latent representations. The major difference is that the autoencoder is trained via the joint minimization of self-reconstruction error defined as the Bernoulli cross-entropy error between input and output (*Empirical cost*), as well as the sum of square error (SSE) between neighbors of input and output (*Regularizer*).

In contrast to LapPLSI, LTM and DTM, our method can provide an explicit parametrized embedding mapping *y* = *f*_*Θ*_(*x*) for extracting the latent semantic representation of new test documents via the estimated parameters of an encoder. In addition, because selected neighbors must have the same class label as the input data, class information is used in our method implicitly, thus enabling LEAE to capture the discriminative structure simultaneously. Finally, we view the activating features, including the connected weights of the hidden neural, as a synthetic document and investigate those words with the strongest activating connections. Some understandable combination of words is detected, which can improve semantic comprehension of latent representation. We provide empirical evidence on two different lengths using a widely used dataset (20 newsgroups and Web-snippets) and demonstrate the superiority of LEAE compared to comparative techniques.

## Related Work

Laplacian eigenmaps (LEs) have demonstrated that manifold property can be a discrete approximation by the nearest neighbor graph of scattered observation points[21]. Consequently, based on PLSA, LapPLSI, LTM, and DTM use manifold structure information by incorporating graph regularization on the original objective function of PLSA. As a result, the topic distribution *P*(*z*_*k*_*|d*_*i*_) can assign more similar latent representation to documents that are located closely on the manifold. The graph regularization can be defined as follows:

$$\sum_{i,j}W_{ij}Dist\left(y_{i},y_{j}\right),$$ (1)

where *y* is latent representation and *Dist(*.*)* is a function used to measure distance in the latent representation space. *W*_*ij*_ is the edge weight of the nearest neighbor graph between instances *i* and *j* [25].

The major difference between these models is the definition of *Dist(*.*)*. LTM adopts the Kullback-Leibler (KL) divergence as the distance in the latent representation space, whereas DTM and LapPLSA define *Dist(*.*)* as the Euclidean distance. Additionally, to inherit the full discriminating power from the global manifold structure, DTM goes further to consider negative relationships over documents[25]. Such models address out-of-sample data optimally through an inclusive approach, which must reconstruct the nearest neighbor graph of new data and fit the model again. This requirement is necessary because LEs cannot provide a specific mapping relationship from the input space to the latent space for out-of-sample data. Repeating the entire modeling process is inefficient and limits the usefulness of these methods in practical usage.

The basic autoencoder, also called autoassociators[29], is a one-hidden-layer multi-layer perceptron (MLP) aiming to reconstruct the original input as correctly as possible. Therefore, the expected output of the autoencoder is the input itself. It consists of an encoder *f*_*θ*_, which encodes an input vector *x*∈ℝ^*d*^ to a latent representation *y* = *f*_*θ*_(*x*)∈ℝ^*k*^, as well as a decoder *g*_*θ'*_, which decodes *y* back to the input space $\hat{x}=g_{\theta \prime }(y)$ as the reconstruction of *x*. The parameters *θ*, *θ*' are learned via a back-propagation algorithm to minimize reconstruction error (RE) over a dataset. Based on the basic autoencoder, the DAE[26], CAE[27] have been proposed by adding an additional regularization term, which aims to obtain a better latent representation of observation data concentrated in the vicinity of a smooth lower-dimensional manifold. DAE corrupt the input stochastically and learn to recover the uncorrupted input from the corrupted data[30]. During denoising training, DAE may capture the manifold structure of the input distribution. CAE adds the Frobenius norm of the encoder’s Jacobian to the objective function of the basic autoencoder, and thus, the results are less sensitive to the input despite being sensitive to variations along the high-density manifold[8]. However, DAE and CAE focus purely on self-reconstruction without explicitly modeling the data relationship. To address this issue, Generalized Autoencoder (GAE)[31] first model the data relationship by computing relational weights between each instance of *x*_*i*_ and other data {*x*_*j*_, *x*_*k*_…} and then use the encoded latent representation to reconstruct other relational instances with relational weights while ignoring self-reconstruction. The DAE, CAE and GAE disregard valuable class label information.

## Methods

**Fig 1:**
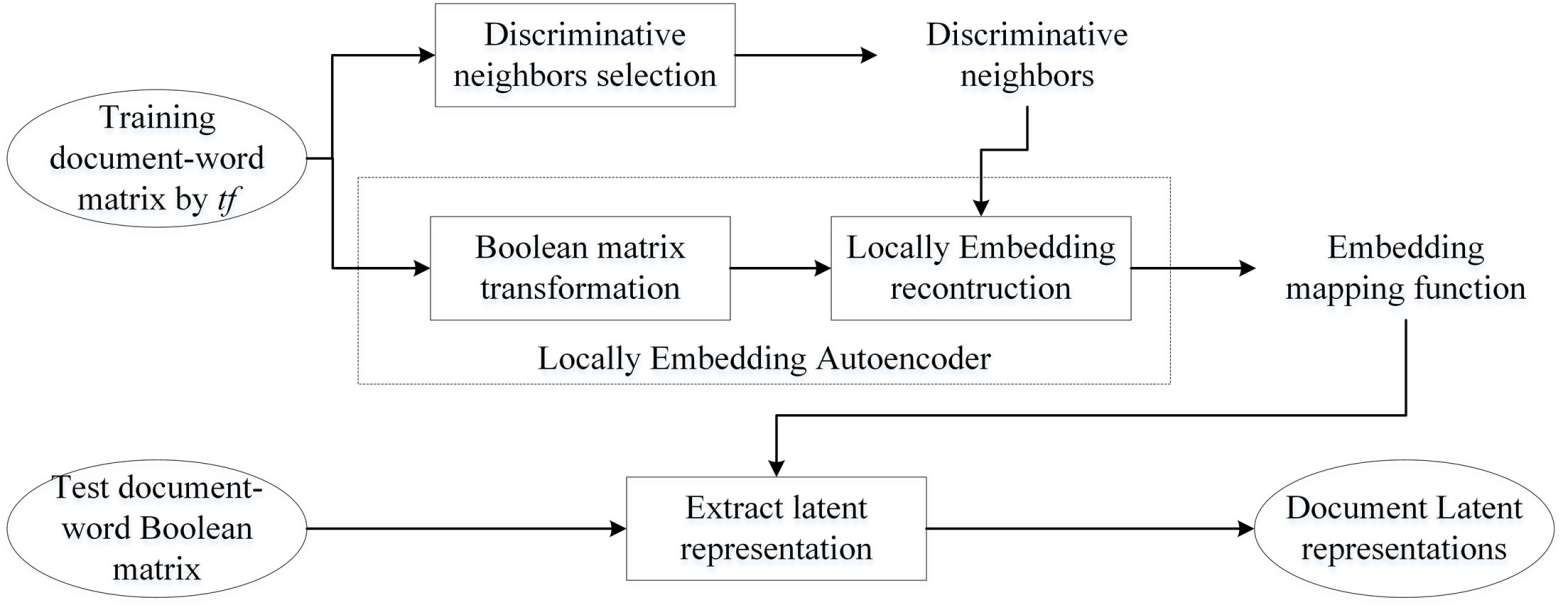
Block diagram of the locally embedding autoencoder.

The block diagram of our approach is shown in Fig 1, and the main idea is as follows: motivated by manifold hypothesis that assume natural observations in high-dimensional spaces are likely to generate from a low-dimensional manifold [32], we assume that document representation in the observation space is generated from a smooth, low-dimensional manifold and wish to recover document representation in the latent low-dimensional space based on observation data via an explicit embedding mapping. Specifically, supposing that such latent document representation is strongly dependent on its neighbors, from the view of bag of words model, we first represent each document in the forms of a count vector, and select the discriminative neighbors set with Euclidean distance in the observation space. Then, the autoencoder is trained by jointly minimizing the Bernoulli cross-entropy error between the input and output (*Empirical cost*), as well as the SSE between the neighbors of the input and output (*Regularizer*). Finally, the encoder *y* = *f*_*θ*_ (*x*) can play the role of an explicit parametrized embedding mapping function in extracting the latent representation of new test documents.

### Discriminative neighbor selection

**Fig 2:**
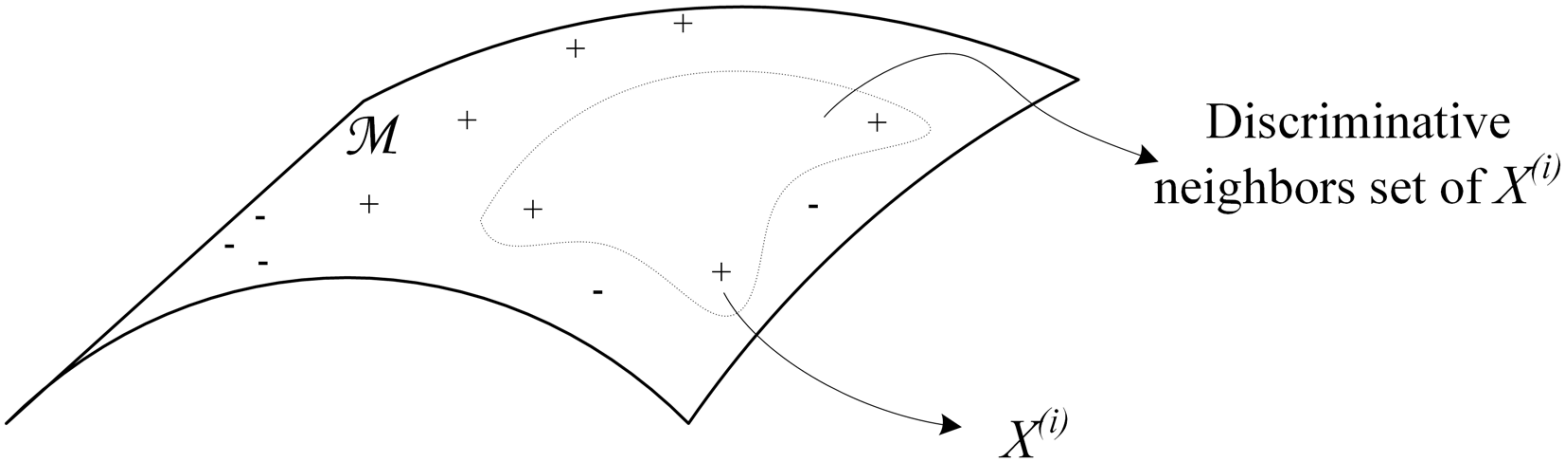
Geometrical representation of discriminative neighbors for *X*^*(i)*^.

In mathematics, manifold is interpreted as a topological space that resembles Euclidean space near each point, this can be referred as locally Euclidean or local consistency. From the view of manifold, manifold is locally Euclidean in that every point has a neighborhood, called a chart, homeomorphic to an open subset of R^n^. The coordinates on a chart allow one to carry out computations as though in a Euclidean space[33]. Therefore, we first find the nearest neighbors with the Euclidean distance and then select the nearest neighbors with the same category label as those discriminative neighbors, which provide a local discriminative geometrical structure. Given a training document-word matrix{*X*^(1)^, …*X*^(i)^, …*X*^(m)^}, let *X*^*(i)*^ = [*x*_*1*_, *…x*_*j*_, *…x*_*d*_] be the document vector, a *d*-dimensional vector in the word space ℝ^*d*^, where *d* is the size of vocabulary, and *x*_*j*_ is a measurement of term frequency (*tf*) of the word *j*. Specifically, to prevent a bias towards longer documents, we adopt a ratio of a term’s occurrences in a document and the sum of term frequency of any word within the same document. Let *C*^*(i)*^ be the class label of *X*^*(i)*^ and *S*^*(i)*^ = {*X*^*(i*,*1)*^,…*X*^*(i*,*K)*^} be a set of discriminative neighbors. This relationship is illustrated in Fig 2, where “+”and “-” denote documents with different labels. For document *X*^*(i)*^ located on a manifold *M*, the area surrounded by the dotted line contains its discriminative neighbors. Table 1 is the procedure used to determine the set of neighbors in our method.

**Table 1:** Pseudo-code of Algorithm 1.

**Algorithm 1** Select the discriminative neighbor set
**Input**: *K* is the nearest neighbor numbers, and *D* = {(*X*^*(1)*^, *C*^*(1)*^), …(*X*^*(i)*^, *C*^*(i)*^), …(*X*^*(n)*^, *C*^*(n)*^)}.
**Output:** discriminative neighbor set *S*^*(i)*^
**For** each instances (*X*^*(i)*^, *C*^*(i)*^)
Compute its Euclidean distance to some other document vector *d*(*X*^*(i)*^, *X*^*(j)*^), subject to *C*^*(i)*^ = *C*^*(j)*^ and (*i ≠ j*);
Rank *d*(*X*^*(i)*^, *X*^*(j)*^) and select the *K*th nearest instances to *X* ^*(i*,*K)*^
**End for**

### Locally embedding the autoencoder

To find an explicit parametrized embedding mapping for recovering document representation in the latent space based on observation data, we employ the autoencoder to extract the latent representation by the encoder and then reconstruct the document representation in the observation space by a decoder. For a document *X*^*(i)*^ = [*x*_*1*_,…, *x*_*d*_], let *Y*^*(i)*^ = [*y*_*1*_,…, *y*_*h*_] be the latent representation of *X*^*(i)*^, which is a *h*-dimensional vector in latent space ℝ^*h*^, where *h* is a quantity of dimensions, and *d* > *h*. Let *Z*^*(i)*^ = [*z*_*1*_,…, *z*_*d*_]denote the reconstruction representation of *X*^*(i)*^, where *z*_*d*_ denotes the occurrence probability of the *d*th word in the vocabulary.

#### Boolean matrix transformation

For a document *X*^*(i)*^ = [*x*_*1*_,…, *x*_*d*_], we only focus on whether one word occurs, ignoring frequency, because high-frequency words may not always reflect their importance to the document. Here, we suppose that the occurrence of a word is related to binary random variables, transforming the document-matrix into a Boolean matrix. Specifically, for *i*th word in the vocabulary, we have

$$x_{i}=\left\{ \begin{array}{l} 1,\;if\;x_{i}>0,or\;word\;i\;occur\\ 0,\;otherwise \end{array}\right.$$ (2)

Therefore, we obtain a binary vector to represent each document and use the LEAE to carry out the reconstruction process. It should be emphasized that the Boolean matrix transformation is performed only when a document vector weighted by *tf* is the input, and the discriminative neighbors of the input will not be transformed.

#### Locally embedding reconstruction

**Fig 3:**
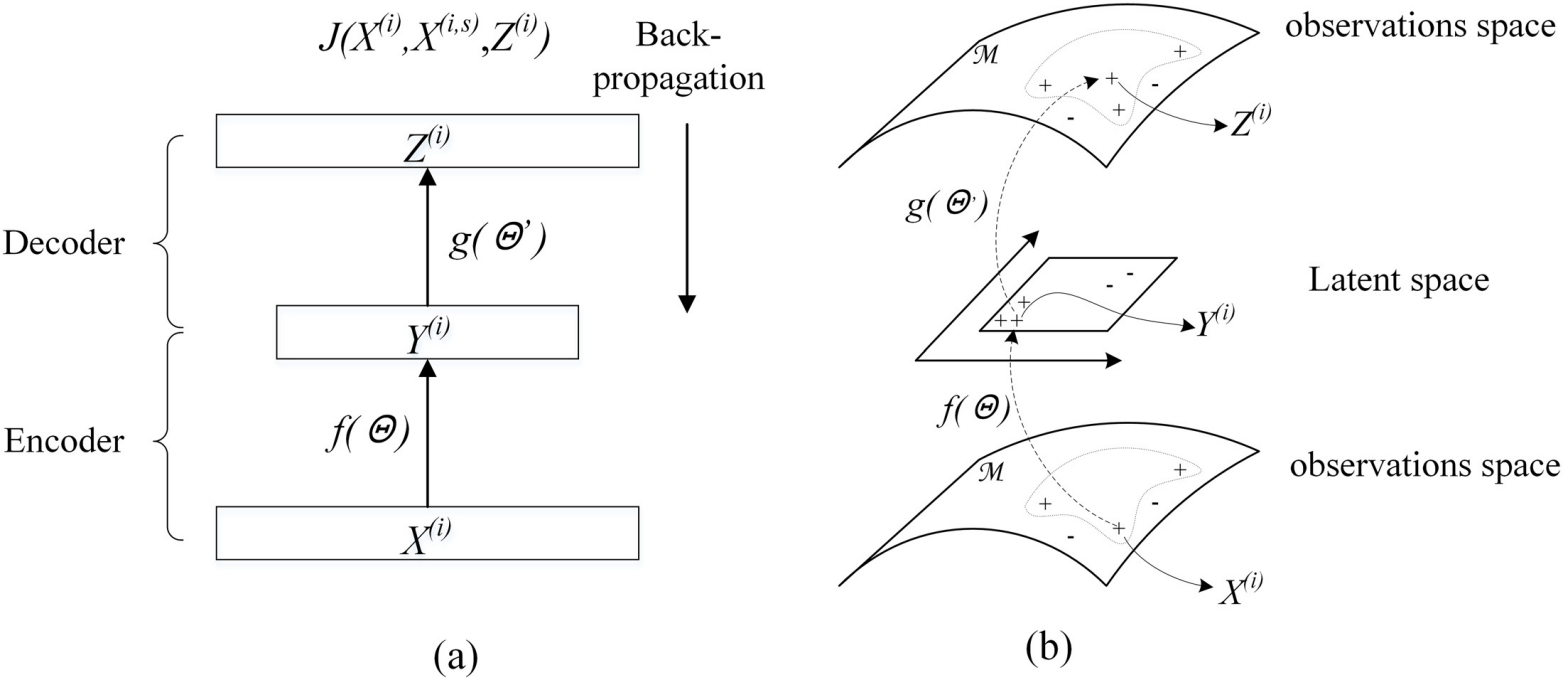
(a) shows the architecture of the autoencoder, whose input and output layers have the same size despite having smaller hidden layers. The bottom network performs the role of an encoder, whereas the top network performs the role of a decoder; (b) geometrical representation of the LEAE.

As illustrated in Fig 3(a), the reconstruction process of input document *X*^*(i)*^can be defined as follows:

- Firstly, latent representation can be generated via encoder *Y*^(i)^ = *f*_*θ*_ (*X*^(i)^) = *σ*(*Y* | *X*^(i)^, *W*, *b*);
- Next, the decoder *Z*^(i)^ = *g*_*θ'*_ (*Y*^(i)^) = *σ*(*Z* | *Y*^(i)^, *W*^*T*^, *c*) is responsible for the allocation of word occurrence under *Y*^*(i)*^;

Consequently, the parametric form of *Z*^(*i*)^ is

$$Z^{\left(i\right)}=g_{\theta \prime }\left(f_{\theta }(X^{\left(i\right)})\right)=\sigma \left(W^{T}\sigma \left(WX^{\left(i\right)}+b\right)+c\right).$$ (3)

where *σ* is the element-wise logistic sigmoid *σ*(*a*) = (1+exp{*a*})^-1^, *W* is the weight matrix of the encoder, which connects the input layer and hidden layer, and *W*^*T*^ is the weight matrix of the decoder, which is shared with the encoder (tied weights); *b* is bias of the hidden layer; and *c* is the bias of the output layer.

In contrast to the basic autoencoder, we suppose that latent representation depends on the input as well as the discriminative neighbors of the input. That is, a good latent representation should be a likely encoding of the data permitting approximation of word occurrence in each document and its neighbors with high probability. Therefore, we define the reconstruction error with the Bernoulli cross-entropy error between the input and output (*Empirical cost*) and the SSE between neighbors of the input and output (*Regularizer*). The reconstruction error is expressed as follows:

$$J\left(X^{\left(i\right)},S^{\left(i\right)};\theta \right)=\left[-\sum_{j=1}^{d}\left(X_{j}^{\left(i\right)}\text{log}\left(Z_{j}^{\left(i\right)}\right)+\left(1-X_{j}^{\left(i\right)}\right)\text{log}\left(1-Z_{j}^{\left(i\right)}\right)\right)\right]+\frac{\lambda }{2K}\sum_{j=1}^{K}{\left\|X^{\left(i,j\right)}-Z^{\left(i\right)}\right\|}^{2}.$$ (4)

*K* is the size of the discriminative neighbors set, and *X*^*(i*,*j)*^ refers to an element of the discriminative neighbors set in relation to *X*^*(i)*^. *d* indicates the size of *X*^(*i*)^ and *Z*^(*i*)^, and *λ* is a non-negative hyper-parameter that has control over the trade-off between *Empirical cost* and *Regularizer*.

The LEAE defines an artificial document as the target output by incorporating the distinctive word co-occurrence patterns within discriminative neighbors. Through minimizing the joint error over *Empirical cost* and *Regularizer*, the autoencoder can yield an output close to the geometrical centroid of discriminative neighbors and the input (see Fig 3(b)). In other words, our method finds an explicit parametrized embedding mapping that varies smoothly along with neighbors of the input data and tends to generate similar representation to nearby points. Therefore, LEAE can better capture semantic structure in the document manifold.

#### Parameter learning

To constitute an explicit parametrized embedding mapping, we must find the value of parameter vectors *θ* (*W*, *b*, and *c*) to minimize *J*(*θ*; *X*^*(i)*^, *S*^*(i)*^). The parameter learning problem can be solved by training this regularized neural network with a mini-batch stochastic gradient descent (SGD). The partial derivatives computation with respect to the input *X*^*(i)*^ is the key step of parameter learning. We first provide the following notation for the partial derivative computation in Table 2:

**Table 2:** Some notations for the partial derivative computation.

*d*	size of the input and output
*h*	size of the hidden units
*x*_*l*_, *l∈{1*,*2*,*…*,*d}*	value of the *l*th input
*x*_*l*_^*(j)*^	value of the *l*th *j* nearest neighbors of input
*z*_*l*_, *l∈{1*,*2*,*…*,*d}*	value of the *l*th output
*y*_*i*_, *i∈{1*,*2*,*…*,*h}*	value of the *j*th hidden unit
*W*_*ij*_	connecting weight between the *i*th hidden unit and *j*th input and connecting weight between the *i*th hidden unit and *j*th output
*b*	bias of the hidden layer
*c*	bias of the output layer
*θ*	any parameters to be estimated
*λ*	non-negative regularization hyper-parameter
*n*	size of each batch training
*J*(*θ*; *X*^*(i)*^, *S*^*(i)*^)	reconstruction error for given input *X*^*(i)*^

The *J*(*θ*; *X*^*(i)*^, *S*^*(i)*^) can be expressed as follows:

$$J(\theta ;X^{\left(i\right)},S^{\left(i\right)})=\left[-\sum_{l=1}^{d}\left(x_{l}\text{log}\left(z_{l}\right)+\left(1-x_{l}\right)\text{log}\left(1-z_{l}\right)\right)\right]+\frac{\lambda }{2K}\sum_{j=1}^{K}\sum_{l=1}^{d}{\left\|z_{l}-x_{l}^{\left(j\right)}\right\|}^{2}.$$ (5)

Therefore, we have

$$\frac{\partial J}{\partial \theta }=\sum_{l=1}^{d}\frac{z_{l}-x_{l}}{z_{l}(1-z_{l})}\frac{\partial z_{l}}{\partial \theta }+\frac{\lambda }{K}\sum_{k=1}^{K}\sum_{l=1}^{d}\left(z_{l}-x_{l}{}^{\left(k\right)}\right)\frac{\partial z_{l}}{\partial \theta }.$$ (6)

According to the decoder and the logistic sigmoid function, we have

$$z_{l}={\left(1+\text{exp}\left\{b_{l}+\sum_{i=1}^{h}W_{il}y_{i}\right\}\right)}^{-1}.$$ (7)

$$\frac{\partial z_{l}}{\partial W_{ij}}=\left(W_{il}y_{i}(1-y_{i})x_{j}+1_{l=j}y_{i}\right)(1-z_{l})z_{l}.$$ (8)

$$\frac{\partial z_{l}}{\partial b_{i}}=W_{il}y_{i}\left(1-y_{i}\right)\left(1-z_{l}\right)z_{l}.$$ (9)

$$\frac{\partial z_{l}}{\partial c_{j}}=\left\{ \begin{array}{ll} \left(1-z_{l}\right)z_{l} & if\;l=j\\ 0 & if\;l\neq j \end{array}\right..$$ (10)

Finally, from these equations and Eq 6,

$$\begin{array}{ll} \frac{\partial J}{\partial W_{ij}}= & \frac{\lambda }{K}\sum_{k=1}^{K}x_{j}y_{i}\left(1-y_{i}\right)\left(z_{l}-x_{l}{}^{\left(k\right)}\right)+x_{j}y_{i}\left(1-y_{i}\right)\sum_{l=1}^{d}\left(1-z_{l}\right)z_{l}\left(z_{l}-x_{l}{}^{\left(k\right)}\right)W_{il}\\ & +(z_{j}-x_{j})y_{i}+y_{i}(1-y_{i})x_{j}\sum_{l=1}^{d}\left(z_{l}-x_{l}\right)W_{il}. \end{array}$$ (11)

$$\frac{\partial J}{\partial b_{i}}=y_{i}\left(1-y_{i}\right)\sum_{l=1}^{d}\left(z_{l}-x_{l}\right)W_{il}+\frac{\lambda }{K}\sum_{k=1}^{K}y_{i}\left(1-y_{i}\right)\sum_{l=1}^{d}\left(1-z_{l}\right)z_{l}\left(z_{l}-x_{l}{}^{\left(k\right)}\right)W_{il}.$$ (12)

$$\frac{\partial J}{\partial c_{j}}=\left(z_{l}-x_{l}\right)+\frac{\lambda }{K}\sum_{k=1}^{K}\left(1-z_{l}\right)z_{l}\left(z_{l}-x_{l}{}^{\left(k\right)}\right).$$ (13)

The pseudo-code of the parameter learning algorithm is shown in Table 3.

**Table 3:** Pseudo-code of Algorithm 2.

**Algorithm 2** Parameter learning for LEAE
**Input:** The training set ${\left\{X^{\left(i\right)}\right\}}_{i=1}^{m}$.
**Output:** the parameter of embedding mapping *W*, *b*, *c*;
Randomly shuffle the training set. Randomly initialized *W*, *b*, *c*
**For** each *epoch*
**For** each *batch* instances ${\left\{X^{\left(i\right)}\right\}}_{i=1}^{n}$
Select the discriminative neighbors set of *X*^*(i)*^;
Calculate the activations for the hidden layer and output layer via a feedforward pass;
Compute the partial derivatives in regard to the input as Eqs 11, 12 and 13
Compute: $\Delta W=\sum_{n,i,j}\frac{\partial J}{\partial W_{ij}}$;$\Delta b=\sum_{n,i}\frac{\partial J}{\partial b_{i}}$;$\Delta c=\sum_{n,j}\frac{\partial J}{\partial c_{j}}$
Update: W=W−α(1nΔW);b=b−α(1nΔb);c=c−α(1nΔc)
**End for**
**End for**

## Experiments

Here, we first investigate two common applications of DR (i.e., document clustering and classification) to assess the discriminative performance of the LEAE. We compare the LEAE with several unsupervised and supervised state-of-the-art approaches using two widely used text corpora (20 newsgroups and Web-snippets).

- Latent semantic indexing (LSI, unsupervised)[9];
- Supervise latent Dirichlet allocation (SLDA, supervised)[14];
- Locally consistent topic modeling (LTM, supervised)[24];
- Discriminative topic model (DTM, supervised)[25];
- Denoising autoencoder (DAE, unsupervised)[26];
- Contractive autoencoder (CAE, unsupervised)[27];
- Discriminative LDA (DLDA, supervised)[34];
- Latent Dirichlet allocation with belief propagation (LDA-bp, unsupervised)[35];
- The approach using raw word histograms (VSM, unsupervised)

In addition, we also assess whether the LEAE can obtain discriminative representations of documents without considering the class labels in Algorithm 1(LEAE with unsupervised, denote LEAE-us). For example, we assume all document belonging to the same class.

### Datasets

The 20 newsgroups corpus is a widely used corpus belonging to 20 related categories. Here, we use the preprocessed version, which does not include cross-posts (duplicates) and newsgroup-identifying headers (Xref, Newsgroups, Path, etc). It includes 18,821 documents and 8,156 distinct words. Web-snippets is a set of search snippets belonging to 8 domains/categories, which are available online [36]. It has 12,340 snippets with 30,338 distinct words. Search snippets are short, sparse and less topic-focused, thus resulting in difficulties related to topic modeling. Table 4 shows some statistical information for those datasets, where *D* is the amount of documents, *W* is the vocabulary size, $\bar{D}$ is the average length of documents, *St*.*Dev* is the standard deviation in document length, *D*_*train*_ is the size of the training set, and *D*_*test*_ is the size of the test set.

**Table 4:** Statistical information of the 2 corpora.

Corpus	*D*	*W*	$\bar{D}$	*St*.*Dev*	*D*_*train*_	*D*_*test*_
20 newsgroups	18,821	8,165	65.29	75.31	12,000	6,821
Web-snippets	12,340	30,338	12.99	3.60	9,000	3,340

### Experimental procedure

**Fig 4:**
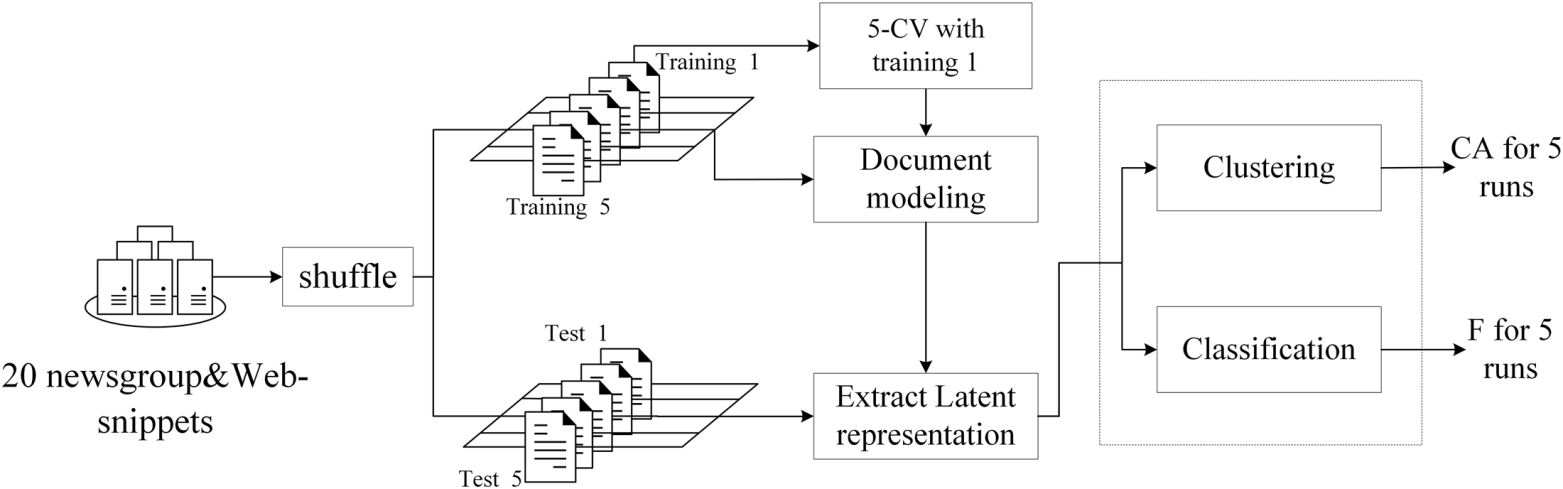
Flow diagram of the experimental procedure.

To obtain a fair experimental performance, we conducted 5 runs for the 2 datasets. First, we randomly shuffled both corpora 5 times and divided each corpus into 2 parts. In the 20 newsgroups corpus, we saved 6,821 documents for test purposes and trained LEAE and other comparative models on the remaining 12,000 documents. In the Web-snippets corpus, we saved 3,340 snippets for a test and trained different models on the remaining 9,000 documents. Next, in the first run, the training set was used for the training model by 5-fold cross validation (CV). The optimal parameters of all approaches were obtained based on performance. Finally, another 4 runs were conducted on the remaining 4 shuffled datasets with the same chosen parameters. Fig 4 is the flow diagram representing this experimental procedure.

For the LEAE and LEAE-us, we adopted the mini-batch SGD to minimize Eq 4 with the optimal hyper-parameters obtained after 5-CV (*n* = 100, *α* = 1.2, *epoch* = 30, *K* = 7, *λ* = 100). Finally, we utilized the explicit parametrized embedding mapping to extract the latent representation. In particular, for LEAE, we treat the domains of Web-snippets as categories to select the discriminative neighbors set.

For the LTM and DTM, we fixed 20 neighbors for the construction of neighborhood graphs, and selected *λ* = 1,000, indicating that graph regularization plays a more important role when learning topic distribution. In addition, we used class label information to compute the similarity matrix by adding an edge between two documents related to the same class and removing an edge between documents related to different classes[24]. In contrast, for the construction of the dissimilarity matrix $\bar{W}$, an edge should be added for documents related to different classes and removed for documents related to the same class. To address the limitation on handling a previously unseen document, we employ inclusive approaches that rebuild similarity and dissimilarity matrices with out-sample documents, retraining the model based on these matrices[25]. Since the graph regularization of the LTM and DTM is based on LE algorithms, which cannot provide a specific mapping function from the manifold to the output embedding[37]. This step gives both models an unfair advantage over other models.

We trained LDA with belief propagation under the same hyper-parameters setting[35] *α*, *β* = 1*e* − 2, and *max iterations* = 500. We also carried out a comparison with LSI using a Matlab toolbox. For SLDA, we trained the topic model under the following setting: *var max iter* = 100; *var convergence* = 1*e*-3; *em max iter* = 300; *em convergence* = 1*e*-4; *L2 penalty* = 1*e*-2; *alpha* = 1*e*-2; *initialization* is "random". For DLDA, we obtained the parameters using a Matlab toolbox with random initialization.

### Experimental results

#### Discriminative performance evaluation of clustering

We employed K-means to group test documents formalized in the latent representation space. As a common technique for statistical data analysis, K-means automatically groups instances according to their distances in the representation space, which can reveal the intrinsic structure of a corpus. We fixed the number of clusters at 20 and explored several numbers of topics (50, 100, 150, 200, 250 and 300). The clustering results for the 5 shuffled datasets were evaluated for clustering accuracy (CA). Given document *X*^(i)^, let *C*_*i*_ be the assigned cluster id and *S*_*i*_ be the original label. The computation of CA is as follows[24]:

$$CA=\frac{\sum_{i=1}^{N}\delta (S_{i},map(C_{i}))}{N}.$$ (14)

where *N* indicates the size of the test documents and *map*(*C*_*i*_) matches *C*_*i*_ to equivalent document labels. The determination of optimal mapping can refer to the Kuhn-Munkres algorithm[38]. *δ*(*x*, *y*) is *delta* function defined as follows:

$$\delta \left(x,y\right)=\left\{ \begin{array}{l} 1,\;if\;x=y\\ 0,\text{ otherwise} \end{array}\right..$$ (15)

**Fig 5:**
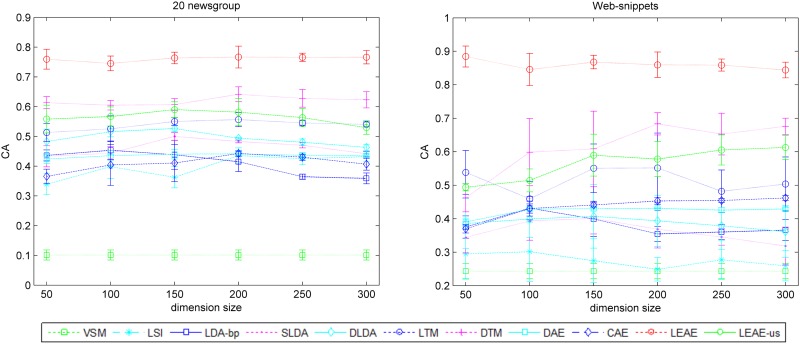
CA of different models on both the 20 newsgroup (left) and web-snippet (right) datasets, with each point consisting of a mean value as well as standard deviations.

Fig 5 demonstrates the average performance of several methods over 5 runs. As shown in the results, several methods that extract latent representation outperform VSM. Moreover, compared with approaches that suppose that the document is located in the Euclidean space (i.e., LSI, LDA-bp, SLDA, and DLDA), LEAE, DTM and LTM can still achieve better performance, whereas CAE and DAE show slight improvements over LSI and LDA-bp but lag behind DLDA and SLDA. Among all of these methods, LEAE improves the CA by up to 15% compared to the other methods, and the average performance is also steadier for both corpora when compared with other manifold-based methods. In particular, for the web-snippets, our method still achieves satisfactory results even when the dimension number is 50. In addition, the LEAE-us show the superiority to other unsupervised approaches, furthermore, LEAE-us is even better than some supervised approaches, such as LTM and SLDA. This evidence demonstrate LEAE with unsupervised setting is also able to enhance clustering performance to some extent.

Moreover, to analyze discriminative performance, t-Distributed Stochastic Neighbor Embedding (t-SNE)[39] was adopted to visualize the 2D embeddings of latent representations generated from different approaches.

**Fig 6:**
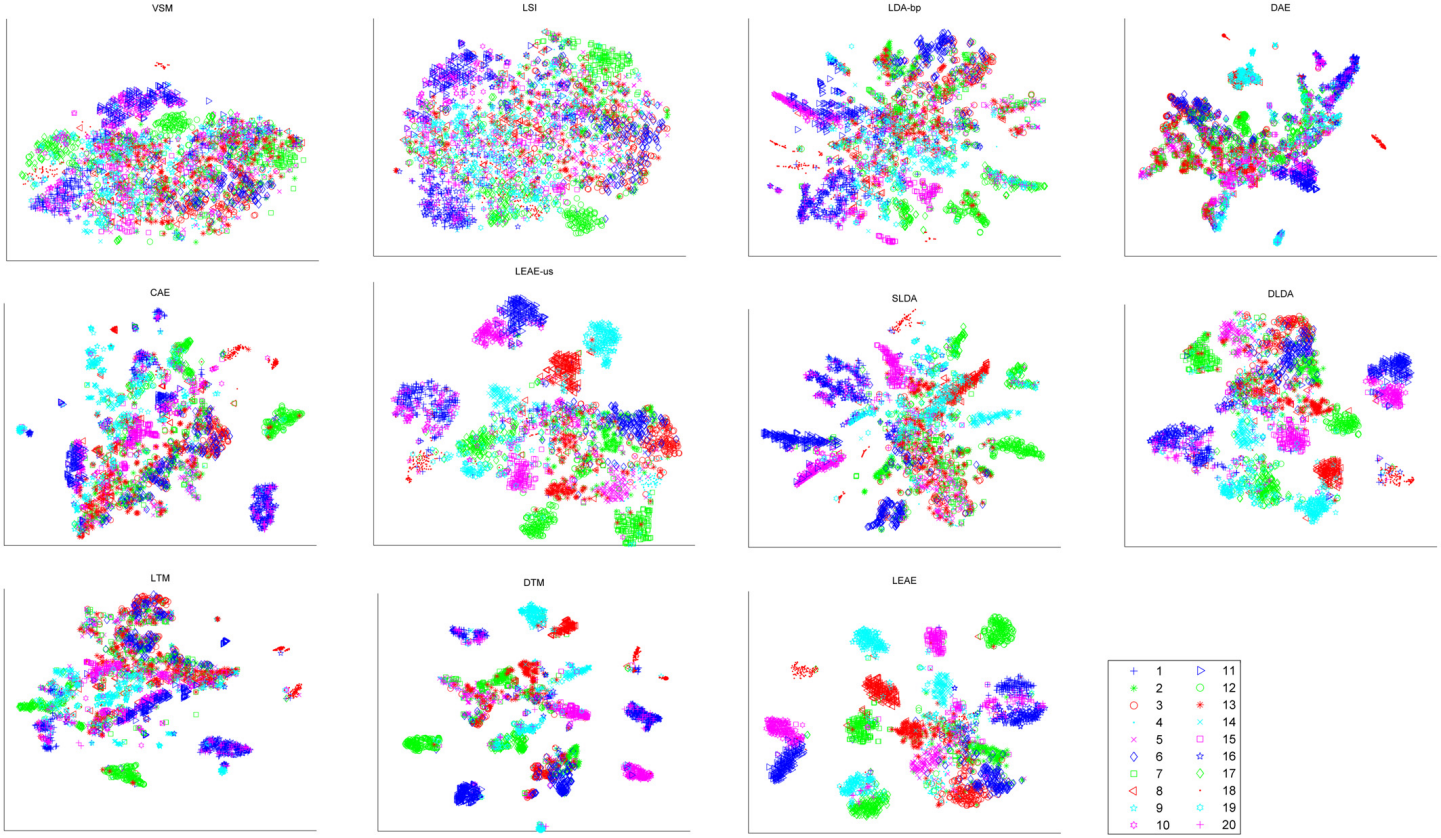
2D embeddings of the latent representations on the 20 newsgroups.

**Fig 7:**
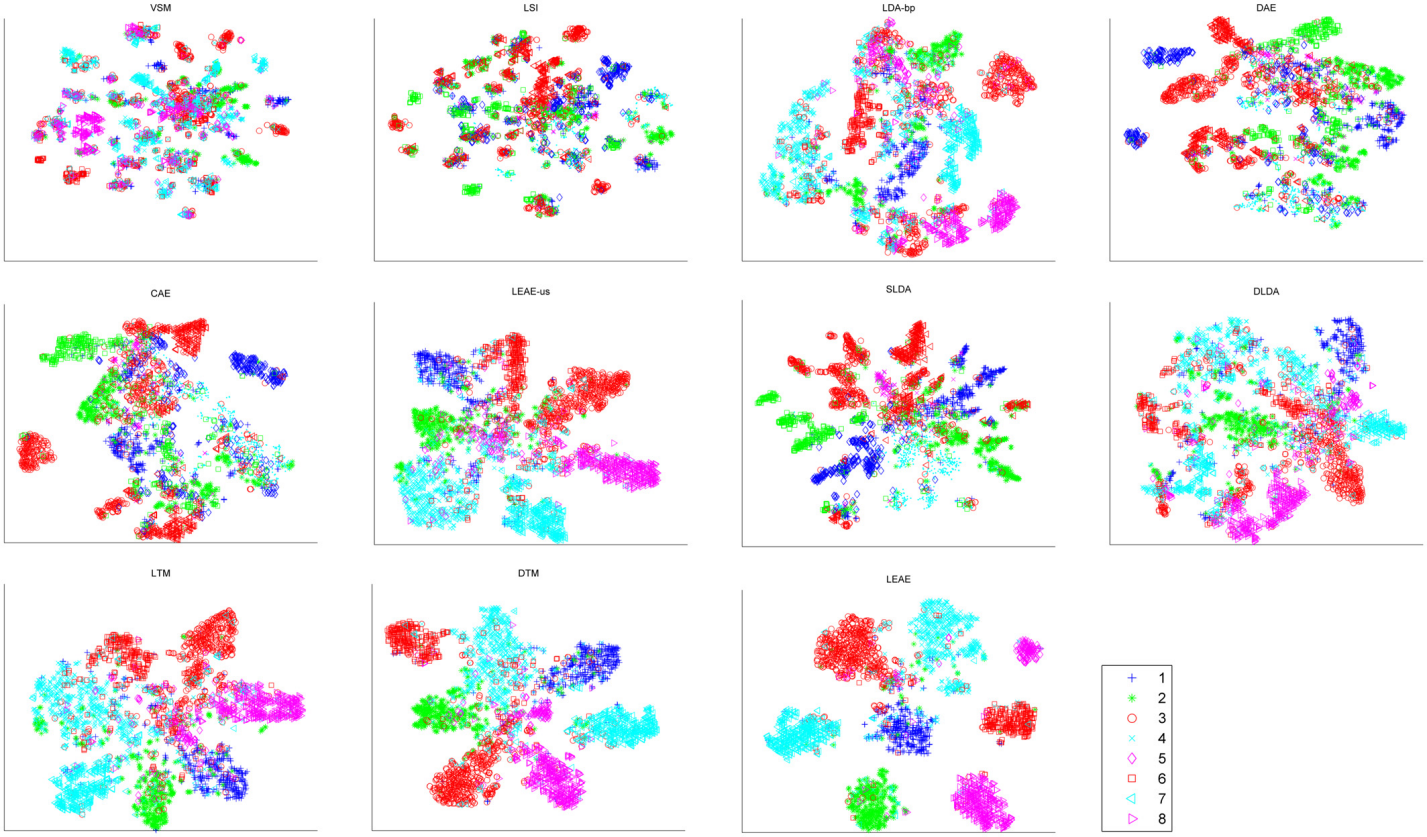
2D embeddings of the latent representations of web-snippets.

Figs 6 and 7 present scatter diagrams of the 2D embeddings of latent representations over the 2 corpora. Each dot indicates a document and each marker denotes a class. The evidence shows that our method provides more separable representation in the 2D embedding space than the other methods.

#### Discriminative performance evaluation of classification

In this section, we further compare the influence of discriminative power provided by several models in a supervised setting. We obtained latent representation of the 5 shuffled test sets and randomly divided it into 2 equal parts. One is applied for test purposes, and the other is used to train the classification model of 1-nearest neighbor (1-NN) and the support vector machine (SVM), respectively. We implemented the classification framework based on WEKA, which provided several popular classification algorithms. In this paper, we used “lazy.IB1” for 1-NN, but for SVM, we employ publicly available java code “LIBSVM”, which could be easily executed by WEKA. In particular, we achieved the classification model by incremental training by testing 50% and training 10%, 30%, and 50%. Because the two corpora contain multiple categories, we used the weighted *F*-measure $\bar{F}$ to estimate the accuracy of the classification model, which is calculated as follows:

$$\bar{F}=\frac{\sum_{i}c_{i}F_{i}}{C}.$$ (16)

where *c*_*i*_ is the proportion of instances in test set categories *i* and *C* is the size of the test set. *F*_*i*_ is the *F*-measure of categories *i*, which can be calculated based on the precision *P*_*i*_ and recall *R*_*i*_. The *P*_*i*_, *R*_*i*_ and *F*_*i*_ are defined as follows:

$$P_{i}=\frac{\left|\left\{relevant\;documents\right\}\right|\cap \left|\left\{retrieved\;documents\right\}\right|}{\left|\left\{retrieved\;documents\right\}\right|},$$ (17)

$$R_{i}=\frac{\left|\left\{relevant\;documents\right\}\right|\cap \left|\left\{retrieved\;documents\right\}\right|}{\left|\left\{relevant\;documents\right\}\right|},$$ (18)

$$F_{i}=2\cdot \frac{P_{i}\cdot R_{i}}{P_{i}+R_{i}}.$$ (19)

**Fig 8:**
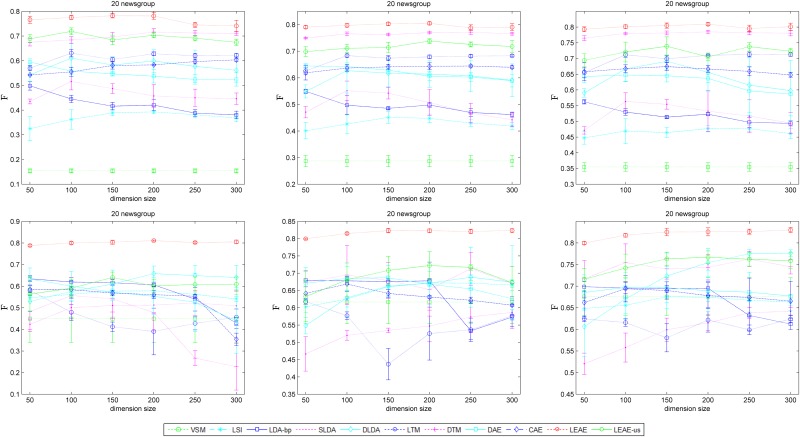
Average classification performance of several models on 20 newsgroups with 1-NN (top) and the SVM (bottom).

$\bar{F}$ represents a weighted average of the classes' *F*-measure, where a higher score indicates better classification performance. Fig 8 is the average $\bar{F}$ and standard deviations after 5 runs on 20 newsgroups with 1-NN (top) and the SVM (bottom). The figure provides the classification performance when training size is 10%, 30% and 50% of the testing size (from left to right).

Fig 8 illustrates that such manifold-based methods (i.e., LEAE, DTM, LTM, CAE and DAE) achieve better $\bar{F}$. Around all methods, LEAE shows more significant improvement of classification performance. Specifically, when the number of training instances equal the number of test instances (right figures), the LEAE increases the average $\bar{F}$ by up to 80.09% (1-NN) and 82.09% (SVM). However, when the ratio of training instances and test instances is 1:5 (left figures), the contribution of our approach is larger than the other remaining methods, at 77.83% (1-NN) and 80.17% (SVM). Another significant advantage of the LEAE is that its performance is consistently the most stable.

**Fig 9:**
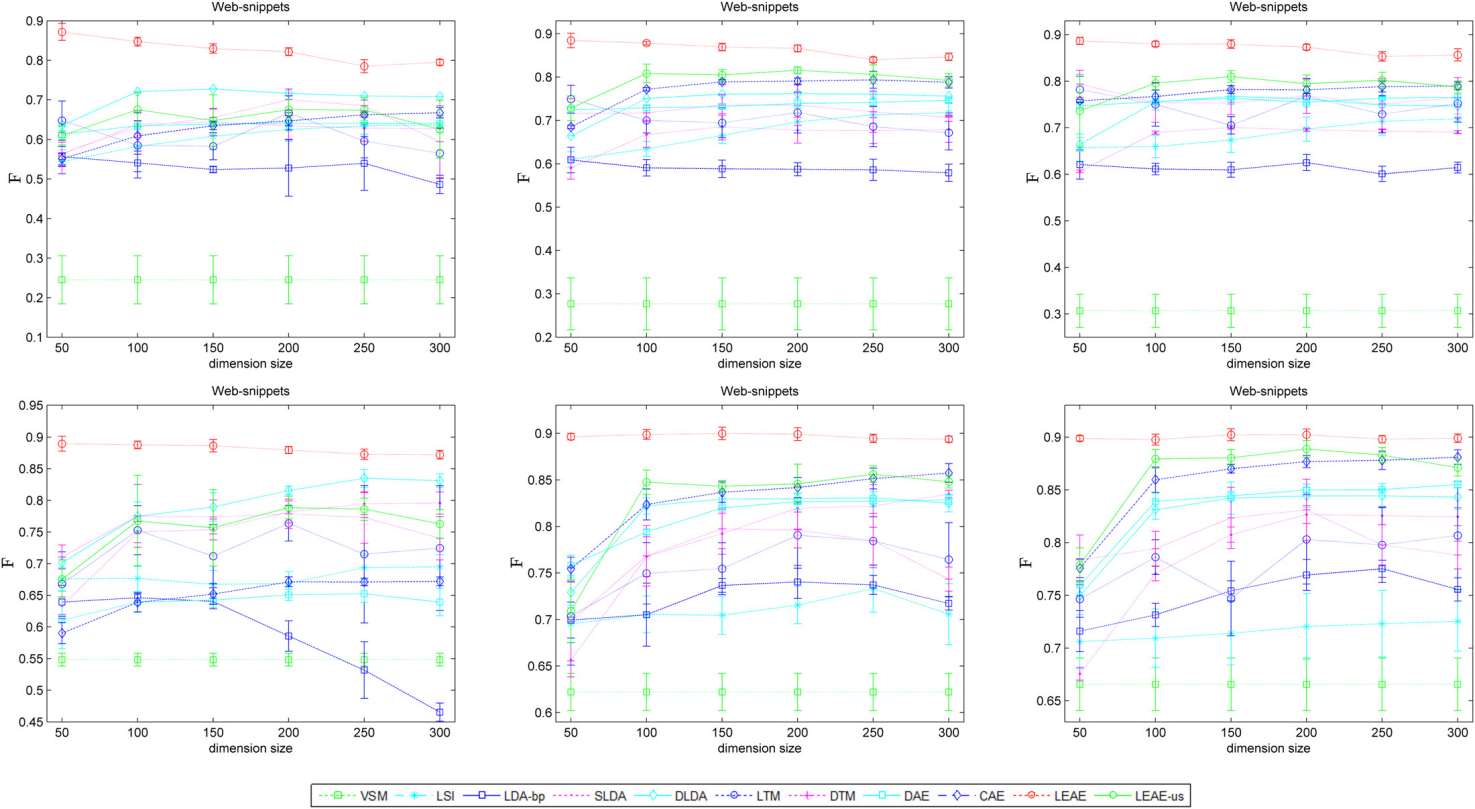
Average classification performance of several models on web-snippets.

Fig 9 provides the average $\bar{F}$ and standard deviations of 5 runs on web-snippets. The figure shows the classification performance when the training size is 10%, 30% and 50% of the testing size (from left to right). The similar evidence shown in Fig 8 demonstrates that the average $\bar{F}$ achieves close to 90%, superior to other models. The performance of the LEAE is the most stable consistently with different training instances. In particular, semi-supervised manifold-based approaches (DTM and LTM) fail to promote average performance, as expected, although they achieve better results than LDA-bp and LSI. Besides, as shown in Figs 8 and 9, LEAE-us achieves satisfactory results and almost beat other methods in some cases.

#### Comprehension of latent representations

To give each dimension of the latent representations a reasonable interpretation, we consider in particular the value of each dimension of latent representation *Y*^*(i)*^ for given document *X*^*(i)*^. In our method, *Y*^*(i)*^ consists of the hidden layer output, whose dimension is the *sigmoid* activation. In detail, the output of unit *j*, denoted as *Y*_*j*_^*(i)*^, depends strongly on the dot product of the input *X*^*(i)*^ and its synaptic weights, denoted as *W*_*j*_ (ignoring the bias term). That is, the synaptic weights associated with that neuron uncover an activating pattern, hence, we denote *W*_*j*_ as activating features. As a synthetic vector or point of latent space, *W*_*j*_ provides the weight of each word, indicating the activating connections between the word space and latent space. Therefore, we treat each row vector of the encoder parameters matrix as a synthetic document and select words with the top 5 activating connections to investigate the meaning of each dimension in latent representations. Table 5 shows partial results and entire results can be found in S1 and S2 Tables under Supporting Information section.

**Table 5:** Top 5 words to topic document of 20 newsgroups and web-snippets (dimension number = 50).

20 newsgroups				web-snippets			
h13	h25	h30	h32	h1	h7	h31	h46
gun	Israel	car	player	health	programming	graduate	sports
weapon	isra	bmw	game	healthy	language	college	football
firearm	hate	driver	season	calorie	java	research	match
arm	kill	auto	bike	food	cache	students	golf
control	armenia	speed	playoff	prevention	memory	harvard	tournament

Table 5 illustrates for 20 newsgroups, the top 5 words from the hidden units 13(h13), 32(h32), 30(h30) and 25(h25) can be understood as topics related to guns, sports, cars and the Middle East. In web-snippets, h1, h31, h46 and h7 refer to health, education, sports and computers. The results show that the activation of features is comprehensible and can efficiently capture some meaningful combinations of words, proving that the semantic structure is not damaged. Consequently, our approach provides a feasible way for latent representation interpretation via such activating features.

In addition, the LEAE can also provide word representation, using the column vectors of the encoder parameter matrix, whose dimension is the weight connections to each hidden unit. Table 6 shows the 5-nearest neighbors of a given wordfor 20 newsgroup, indicating that the LEAE provides meaningful word representations. In particular, batf is abbreviation of Bureau of Alcohol Tobacco and Firearms, and nra indicates National Rifle Association. Table 7 shows the 5-nearest neighbors of a given wordfor web-snippets. We also provide the corresponding result of DTM.

**Table 6:** Top 5 word to topic document of web-snippets (dimension number = 50).

Given word	LEAE	DTM
**god**	jesu bibl christ christian satan	christ satan belief atheist religion
**doctor**	patient disease surgery diet physician	patient medic disease cancer health
**gun**	firearm batf handgun weapon nra	weapon arm control nra clinton
**team**	season score player sport playoff	player season year sport fun
**rocket**	spacecraft payload burster proton aurora	spacecraft payload burster proton aurora
**homosexu**	gay clayton heterosexual optilink molest	men heterosexual gay sex cramer
**bmw**	honda wheel steer ride biker	Car wheel speed honda biker
**graphic**	viewer imag tiff siggraph fractal	display color imag fractal viewer

**Table 7:** Top 5 word to topic document of web-snippets (dimension number = 50).

Given word	LEAE	DTM
**graduate**	research graduated edweek sponsorships e-newsletters	research harvard e-newsletters college students
**weapons**	nuclear detonated invasion republican elections	bombs bomb weapon Iraq military
**militant**	militancy military sparta greekculture city-states	military weapon Iraq republic political
**matlab**	Mathworks matlabcentral developerworks athlon macintosh	server matlabcentral intel operating windows
**income**	consumption consumer gdp revenues investing	market trade economic consumer buy
**import**	export debt investor investing sell	trade business investor sell global
**illness**	illnesses patient webmd infections complications	patient prevention complications healthy medical
**film**	movies movie artist artists imdb	movies artists imdb artist music

#### Discussion

The clustering results demonstrate that our method preserves the inherent manifold structures in a corpus more successfully than other methods. Thus, this method can easily discover more discriminative representation among unseen test documents that are located on manifold. In particular, the experimental results show that unsupervised manifold-inspired methods (i.e., LEAE-us, CAE and DAE) outperform LDA-bp and LSI, confirming that the manifold hypothesis is reasonable. Fig 5 illustrates that our method outperforms other supervised approaches(i.e., DTM, LTM DLDA and SLDA), in that the LEAE utilizes class labels to find determining neighbors. Moreover, among all methods, the LEAE achieves the best result and increases the CA by at least 15%, indicating that the LEAE can measure inherent similarity between documents precisely and help to reveal the intrinsic discriminative geometric structure of a corpus. Similar conclusions can be found intuitively in Figs 6 and 7; the LEAE not only preserves inner-class intrinsic structure but also reduces possible overlap and widens inter-class margins. The reason we interpret this conclusion is that the assumption that the latent representation of each document is strongly associated with its neighbors results in the assigning of similar representation to nearby documents.

Figs 8 and 9 prove that our representation provides better generalization abilities to determinate the semantic label. The classification task is to learn a target function *y = f(x)* and to identify to which categories new observations belong as accurately as possible. Hence, good results indicate that the generalization relationship establishment between class labels and data representation is easy. Based on structural risk minimization, the SVM avoids the local minimum and provides better generalization abilities than other classification algorithms[40]. Although 1-NN is highly restricted in terms of the forms of data distribution in the representation space[41], it is derived from density estimation technology and simply assigns test data to the same class as the nearest point from the training set. The LEAE achieved similar $\bar{F}$ values on 1-NN and the SVM because the procedures that train the autoencoder to reconstruct not only input data but also its discriminative neighbors can actually be interpreted to define an artificial document as the target output of the autoencoder by incorporating the distinctive statistical patterns of word co-occurrence within discriminative neighbors. By minimizing the reconstruction error, the LEAE will capture a likely latent representation of the document that permits an approximation of the word occurrence in all related documents with high probability. This eventuality will allow such documents belonging to the same category to be expressed as similarly as possible in the latent space.

Moreover, the results for web-snippets show that the LEAE can achieve better generalization performance for short documents and that our method is robust in representing documents with sparse word co-occurrence patterns. This is mainly due to the additional reconstruction of each document’s discriminative neighbors, which is an extension of the statistical pattern of words in each text. In contrast, the classification results for web-snippets of the DTM and LTM fail to improve the average performance, as expected. The reason for this result may be that the explicit construction of neighborhood graphs is sensitive to short and sparse search snippets. In addition, LEAE can perform a feedforward pass to extract the latent representation of new documents efficiently, whereas the DTM and LTM must reconstruct similarity and dissimilarity matrices with new data and repeat the entire training process, which is clearly inefficient and also gives this model an unfair advantage. We observe such results because LEs cannot give an explicit mapping relationship to transfer graph regularization to an unseen test document.

Finally, this exploration of the meaning of our latent representation yields inspirational results that some interesting and meaningful combinations of words can be found by activating connections to hidden neural; a process which improves the semantic comprehension of latent representation. However, we have not analyzed how many words are needed to present the meaning of each dimension. Additionally, the determination of discriminative neighbors will be improved by incorporating semantic analysis technology.

## Conclusions and Future Work

In this paper, we proposed a semi-supervised manifold-inspired method, namely, the LEAE, for document representation. In particular, we consider the local discriminative geometric structure of the observation space and use an explicit parametrized embedding mapping to extract the latent representation of documents by minimizing the reconstruction error over the ambient Euclidean space. Consequently, the LEAE can readily assign more discriminative latent representation to unseen test documents located on the manifold. The LEAE is also likely to preserve inner-class instinct structure and reduce inter-class overlap. Additionally, the LEAE can efficiently discover the semantic meaning of activating features that provide understandable latent representation.

In the future, we plan to explore further applications of our model, such as topic visualization and understanding in the context of topic evolution analysis. In addition, it will be a challenge to develop a fast online learning algorithm to estimate parameters in practical applications.

## Supporting Information

S1 Table Top 5 word to topic document of 20 newsgroup (dimension number = 50). (DOCX)

S2 Table Top 5 word to topic document of Web-snippets (dimension number = 50). (DOCX)
