# Supplementary material for: Locally Embedding Autoencoders: A Semi-Supervised Manifold Learning Approach of Document Representation
Source: PLoS One. 2016 Jan 19;11(1):e0146672. doi: 10.1371/journal.pone.0146672 (PMC4718658; doi:10.1371/journal.pone.0146672)
Supplement: S1 Table — (DOCX) [file pone.0146672.s001.docx]

| h1 | h2 | h3 | h4 | h5 | h6 | h7 | h8 | h9 | h10 |
| --- | --- | --- | --- | --- | --- | --- | --- | --- | --- |
| question articl answer read problem | group newsgroup discuss subject  date | medic diseas patient health cancer | price compani market  sell  fund | email  set  list  side design | season team player year  fun | server displai disk window unix | gov  sinc employ nasa  earth | govern  tax libertarian people  state | mhz  cpu  clock faster processor |
| h11 | h12 | h13 | h14 | h15 | h16 | h17 | h18 | h19 | h20 |
| christian religion god  atheist belief | kill  univers armenian turk  soviet | gun weapon firearm arm control | connector  mac  forc  put  float | graphic display color image version | christian jesu  paul word bible | game hockei team playoff espn | power circuit signal radio audio | clinton  gai  relat  bush american | gun  israel clinton waco weapon |
| h21 | h22 | h23 | h24 | h25 | h26 | h27 | h28 | h29 | h30 |
| question answer read  rutger  ask | claim  evid argument truth  true | orbit space earth moon mission | game  team player season  win | israel isra  hate  kill armenia | protect softwar backup user regist | internet bitnet student  comput scienc | monitor keyboard mous screen comput | religion  god  atheist belief believ | car  bmw driver  auto  speed |
| h31 | h32 | h33 | h34 | h35 | h36 | h37 | h38 | h39 | h40 |
| post  person  net  chip  read | player game season  bike playoff | church god cathol christ spirit | card  mail window fax  send | back murder batf come waco | christian jesu  soul word  bibl | men sexual cramer male  sex | program system creat  bit  softwar | opinion  isra  support activ washington | people rutger study interest person |
| h41 | h42 | h43 | h44 | h45 | h46 | h47 | h48 | h49 | h50 |
| window microsoft widget graphic  system | mac  apple  ntsc  intern  chip | turk soviet muslim armenia russian | address phone includ softwar sale | year player team good biggest | mail address work send  sale | run  hit  play baseball  nhl | car  road  drive  speed  driver | bike  ride  motorcycle  bmw  car | window problem install screen program |
