# Supplementary material for: Locally Embedding Autoencoders: A Semi-Supervised Manifold Learning Approach of Document Representation
Source: PLoS One. 2016 Jan 19;11(1):e0146672. doi: 10.1371/journal.pone.0146672 (PMC4718658; doi:10.1371/journal.pone.0146672)
Supplement: S2 Table — (DOCX) [file pone.0146672.s002.docx]

| h1 | h2 | h3 | h4 | h5 | h6 | h7 | h8 | h9 | h10 |
| --- | --- | --- | --- | --- | --- | --- | --- | --- | --- |
| health healthy calorie  food  prevention | political  republic  government  democracy  part | tennis tickets match chicago bulls | pages album  solo  music  art | news sports football fans  soccer | music movie  art  film  arts | programming  language  java  cache  memory | market financial economic trade department | parliamentary  president  presidential  parliament  westminster | party democratic legislative congress  political |
| h11 | h12 | h13 | h14 | h15 | h16 | h17 | h18 | h19 | h20 |
| japanese  chinese  asian  indian  japan | pharmacy  fitness therapy cancer healthcare | political democracy  party government  united | healthy calorie food  nutrition diet | Employment  job  jobs  marketing advertising | releases  sciencedaily  topic  press  definition | cancer human biology  lung bioinformatics | scientific technology engineering scientists applied | culture american japanese western chinese | cup  league soccer fifa  players |
| h21 | h22 | h23 | h24 | h25 | h26 | h27 | h28 | h29 | h30 |
| movie culture film movies music | health  gov  prevention  healthy medical | apple iphone  mobile  itunes  office | movie movies  art  culture imdb | republic military bombs union soviet | health sports football match prevention | discovery research climate ecology  science | computer services market  web  programming | amazon books  dvd  node  utf | edu science  computer  research  theory |
| h31 | h32 | h33 | h34 | h35 | h36 | h37 | h38 | h39 | h40 |
| graduate college research students harvard | genetic  discovery  maxwell  anthropology  exploration | oscar  movie aesthetic literature history | movie oscar  art  fashion health | computer  edu software science theory | wikipedia wiki encyclopedia  page information | degree scholars scholarly master scholarship | business political market trade economic | online  downloads  book  press  books | fashion design guitar designers  violin |
| h41 | h42 | h43 | h44 | h45 | h46 | h47 | h48 | h49 | h50 |
| network server windows intel operating | union  soviet  political republic democracy | weapons bombs weapon bomb  Iraq | music  lyrics  rock  band  pop | theory university natural theoretical physics | sports football match  golf tournament | trade business  global  export  import | health cancer medical diagnosis treatment | sports football team  tennis soccer | online links public  press  download |
